# Human-induced pluripotent stem cells generated from intervertebral disc cells improve neurologic functions in spinal cord injury

**DOI:** 10.1186/s13287-015-0118-x

**Published:** 2015-06-24

**Authors:** Jinsoo Oh, Kang-In Lee, Hyeong-Taek Kim, Youngsang You, Do Heum Yoon, Ki Yeong Song, Eunji Cheong, Yoon Ha, Dong-Youn Hwang

**Affiliations:** Department of neurosurgery, Spine & Spinal Cord Institute, College of Medicine, Yonsei University, Yonsei-ro 50-1, Seodaemoon-gu Seoul, 120-752 Korea; Brain Korea 21 Plus Project for Medical Science, Yonsei University College of Medicine, Yonsei-ro 50-1, Seodaemoon-gu Seoul, 120-752 Korea; Department of Biomedical Science, College of Life Science, CHA University, 335 Pankyoro, Bundanggu Seongnam, 463-400 Korea; Department of Microbiology, College of Medicine, CHA University, 335 Pankyoro, Bundanggu Seongnam, 463-400 Korea; Department of Biotechnology, College of Life Science and Biotechnology, Yonsei University, Yonsei-ro 50-1, Seodaemoon-gu Seoul, 120-752 Korea

## Abstract

**Introduction:**

Induced pluripotent stem cells (iPSCs) have emerged as a promising cell source for immune-compatible cell therapy. Although a variety of somatic cells have been tried for iPSC generation, it is still of great interest to test new cell types, especially those which are hardly obtainable in a normal situation.

**Methods:**

In this study, we generated iPSCs by using the cells originated from intervertebral disc which were removed during a spinal operation after spinal cord injury. We investigated the pluripotency of disc cell-derived iPSCs (diPSCs) and neural differentiation capability as well as therapeutic effect in spinal cord injury.

**Results:**

The diPSCs displayed similar characteristics to human embryonic stem cells and were efficiently differentiated into neural precursor cells (NPCs) with the capability of differentiation into mature neurons in vitro.

When the diPSC-derived NPCs were transplanted into mice 9 days after spinal cord injury, we detected a significant amelioration of hindlimb dysfunction during follow-up recovery periods. Histological analysis at 5 weeks after transplantation identified undifferentiated human NPCs (Nestin^+^) as well as early (Tuj1^+^) and mature (MAP2^+^) neurons derived from the transplanted NPCs. Furthermore, NPC transplantation demonstrated a preventive effect on spinal cord degeneration resulting from the secondary injury.

**Conclusion:**

This study revealed that intervertebral discs removed during surgery for spinal stabilization after spinal cord injury, previously considered a “waste” tissue, may provide a unique opportunity to study iPSCs derived from difficult-to-access somatic cells and a useful therapeutic resource for autologous cell replacement therapy in spinal cord injury.

**Electronic supplementary material:**

The online version of this article (doi:10.1186/s13287-015-0118-x) contains supplementary material, which is available to authorized users.

## Introduction

The advent of induced pluripotent stem cells (iPSCs) opened a new avenue for immune-compatible cell replacement therapy as well as in vitro disease modeling, drug discovery, and toxicity testing [1–4]. Until now, most iPSCs have been generated by using fibroblasts [5], keratinocytes [6], adipose-derived stromal cells [7], and peripheral blood cells [8–10]; however, obtaining somatic cells requires additional painful sampling procedures for patients already suffering from unexpected and sudden trauma such as spinal cord injury (SCI). Therefore, it would be convenient and practical to use tissues removed during emergency surgery after SCI to generate iPSCs for autologous cell replacement therapy.

SCI is caused by spine fracture often resulting from a sports injury, traffic accident, or fall. In any case, the fractured spinal vertebra and intervertebral disc are to be removed by spinal stabilization surgery. Therefore, the dissected tissues may be a useful source for iPSC generation. Furthermore, the tissues and cell types obtained in this case are difficult to achieve with a normal biopsy, providing a unique opportunity for evaluating these cell types as a source for iPSC generation.

Cell therapy using human pluripotent stem cells (hPSCs), such as human embryonic stem cells (hESCs) and iPSCs, is a promising therapeutic approach for patients with SCI. Several reports confirmed the efficacy of hPSC transplantation using animal models of SCI [11].

In this study, we sought to generate iPSCs by using human intervertebral disc cells removed during surgery on patients with SCI. This study reported the first generation of hiPSCs from human intervertebral discs and provided a good example of harnessing “waste” surgical tissue to generate iPSCs for future autologous stem cell therapy for SCI.

## Methods

### Isolation of human disc cells

This study was approved by the Institutional Review Board of Yonsei University. We received all necessary consent from any patients for the use for their tissue samples for the purpose of this study. Dissected disc tissue was washed with 1× phosphate-buffered saline (1×PBS) (Wellgene, Daegu, Korea) and then incubated with collagenase A (Roche, Mannheim, Germany) for 4 h with shaking every hour. The enzyme-treated tissue was filtered through 100-μm mesh (BD Biosciences, Billerica, MA, USA), washed three times with 1×PBS, and finally resuspended in Dulbecco’s modified Eagle’s medium (DMEM)/F12 (Invitrogen, Carlsbad, CA, USA) supplemented with 10 % fetal bovine serum (FBS) (Hyclone, Logan, UT, USA) and 1 % penicillin/streptomycin (P/S) (Invitrogen) for incubation in a humidified chamber (37 °C, 5 % CO_2_).

### Production of retroviruses

Twenty-four hours before transfection, 293T cells (ATCC, Manassas, VA, USA) were seeded onto 10-cm culture dishes (BD Biosciences) at a density of 5×10^4^ cells/cm^2^ and cultured overnight in an incubator (37 °C, 5 % CO_2_). For transfection, 3 μg each of four recombinant Moloney-based retroviral vectors (pMXs; Addgene, Cambridge, MA, USA) expressing human octamer-binding transcription factor 4 (*Oct4*), SRY (sex determining region)-box 2 (*Sox2*), Kruppel-like factor 4 (*Klf4*), and *c-Myc* genes, 2 μg of pGag/Pol (Addgene), and 1 μg of pVSV-G (Addgene) were mixed with Convoy™ Transfection Reagent (ACTGene, Piscataway, NJ, USA) and added to cells of approximately 80–90 % confluence, following the suggestions of the manufacturer. Medium was changed the next morning and collected 2 days later, followed by ultracentrifugation (64,000×*g*, 4 °C, 90 min) for harvesting viruses. Viruses in the pellet were resuspended in 0.1 ml of 1×PBS and used for transduction.

### Generation of induced pluripotent stem cells

Disc cells (approximately 5×10^4^) were seeded in a six-well plate (Thermo Scientific, Hudson, NH, USA) with 3 ml of medium consisting of DMEM, 10 % FBS, and 1 % P/S (all from Invitrogen). After approximately 8–12 h, the disc cells were treated with the retroviral solution with addition of protamine sulfate (5 μg/ml) (Sigma-Aldrich, St. Louis, MO, USA) and subjected to further incubation for 14–16 h. The next day, the cells were washed three times with 1×PBS (Welgene, Gyeongsangbuk-do, Korea), and 3 ml of fresh medium was added. Five days after viral transduction, the disc cells were transferred into 6-cm vitronectin-coated dishes (BD Biosciences) at a density of 1–3×10^4^ cells per dish and were cultured two more days in the disc cell culture medium, followed by further incubation with daily change of the extracelluar matrix-based hPSC medium, as previously established [12]. ESC-like colonies were selected as a clone at approximately 20–25 days and were subjected to expansion.

### Immunocytochemistry

Samples were washed three times with 1×PBS and then fixed for 10 min with 4 % paraformaldehyde (PFA) (Merck, Darmstadt, Germany)/1×PBS. After being washed with 1×PBS three times for 10 min each, the samples were treated with a blocking solution (10 % normal donkey serum/1×PBS) (Jackson ImmunoResearch, West Grove, PA, USA) for 1 h at room temperature (RT). The samples were subjected to consecutive treatments with primary and secondary antibodies for 1 h each at RT. The samples were treated with DAPI (4′,6-diamidino-2-phenylindole) (Vector Laboratories, Peterborough, UK) for 5 min after the secondary antibody treatments.

The primary antibodies used in this study are shown in Additional file 1. The secondary antibodies used in this study include an Alexa Fluor 594 goat anti-mouse IgG (1:500), fluorescein isothiocyanate (FITC) donkey anti-mouse IgG (1:500), Cy3 donkey anti-rabbit IgG (1:500), FITC donkey anti-rabbit IgG (1:500), and FITC donkey anti-chicken IgY (1:500) (all from Jackson ImmunoResearch).

### Alkaline phosphatase staining

Human iPSCs were stained for alkaline phosphatase by using the Alkaline Phosphatase Detection Kit (Sigma-Aldrich) in accordance with the instructions of the manufacturer.

### Reverse transcription-polymerase chain reaction

The total RNA samples were prepared by using a NucleoSpin RNA II kit (Macherey-Nagel GmbH, Dueren, Germany) in accordance with the instructions of the manufacturer. One microgram of total RNA was used to generate cDNAs by using a ReverTra Ace quantitative reverse transcription-polymerase chain reaction (qRT-PCR) Kit (Toyobo, Osaka, Japan). PCR was performed by using an Eppendorf Master cycler gradient (Eppendorf, Hamburg, Germany) with the following conditions: (1) 95 °C denaturation for 2 min, (2) 95 °C denaturation for 40 seconds, (3) 55–63 °C annealing for 30 seconds, and (4) 72 °C polymerization for 1–2 min. Steps (2)–(4) were repeated for 20–35 cycles, depending on the target cDNA, followed by a final polymerization step at 72 °C for 10 min. Primer information used in this study is shown in Additional file 2.

### DNA microarray analysis

Total RNA was isolated by using a NucleoSpin RNA II Kit in accordance with the suggestions of the manufacturer, and 2 μg of total RNA was used for a genome-wide gene expression profiling experiment by using the Illumina array (Illumina, San Diego, CA, USA) at Macrogen (Macrogen, Seoul, Korea). Microarray data were deposited in a public repository such as gene expression omnibus (series record GSE64964).

### DNA methylation analysis

The genomic DNA samples were prepared by using a DNeasy Tissue Kit (Qiagen, Hilden, Germany) in accordance with the instructions of the manufacturer. DNA (2 μg) was treated with an EpiTect Bisulfite Kit (Qiagen) following the instructions of the manufacturer. The promoter areas of the *Oct4* and *Nanog* genes were amplified by PCR, subcloned into a TA cloning vector (RBC Bioscience, New Taipei City, Taiwan), and subjected to sequencing analysis.

### Pluripotency test in vitro

For the in vitro examination of pluripotency, both hESCs and iPSCs were mechanically detached from the plate and cultured in a Petri dish (SPL Life Sciences, Pocheon, Korea) by using the embryoid body (EB) medium (DMEM/F12), 10 % Knockout SR, 1 % non-essential amino acid (NEAA), 0.1 mM β-mercapto ethanol, and 1 % P/S (all from Invitrogen) for 15 days. EBs were attached to Matrigel (BD Biosciences)-coated slides, and EBs were immunostained for representative markers (Additional file 1) of the three germ layers.

### Teratoma formation

For teratoma assay, hPSC colonies were mechanically detached and injected into the testes and muscle of a non-obese diabetic/severe combined immunodeficiency (NOD/SCID) (Orient bio, Gyeonggido, Korea) mouse. Roughly 2×10^6^ hPSCs from a 6-cm dish (BD Biosciences) were injected into a NOD/SCID mouse. The teratomas were dissected 8–10 weeks later and were subjected to hematoxylin-and-eosin (Sigma-Aldrich) staining for histological analysis.

### G-banding analysis

A G-banding karyotype analysis of hESCs and iPSCs was performed in the Sam Kwang Medical Laboratories (Smlab, Seoul, Korea). Cells were treated with 0.03 mg/ml KaryoMAX Colcemid Solution (Invitrogen) overnight and were followed by treating with 0.05 % trypsin (Invitrogen) for 5 min at 37 °C. The trypsin was inactivated by adding DMEM containing 10 % FBS. Pre-warmed hypotonic solution containing equal amounts of 0.4 % potassium chloride (Sigma-Aldrich) and 0.4 % sodium citrate (Sigma-Aldrich) was slowly added to the cells to enhance swelling. Carnoy’s solution (methanol:glacial acetic acid, 3:1) was used to fix the cells for 30 min at RT. The cells were then dropped onto a pre-cleaned slide, and the metaphase spread quality was determined by using a phase-contrast microscope. The slide was hybridized with probes from the SkyPaint DNA Kit (Applied Spectral Imaging, Vista, CA, USA) for human chromosomes for 2 days in a 37 °C humidified chamber. The finished metaphase spreads were visualized and analyzed by using the SkyView spectral imaging system (Applied Spectral Imaging).

### DNA fingerprinting analysis

We performed DNA fingerprinting analyses at the DNA Sequencing Core Facility of Korea Gene Information Center (Seoul, Korea). To verify the genetic relatedness of the iPSC lines to their parent fibroblasts, PCR amplification was performed across discrete genomic regions containing highly variable numbers of tandem repeats. The amplified DNAs were compared by sequencing analysis.

### Induction and culture of neural progenitor cells

For passaging, disc cell-derived induced pluripotent stem cell (diPSC) colonies were mechanically dissected into small fragments with subsequent treatment with 2 mg/ml of collagenase IV (Invitrogen) for 30–60 min. The colony fragments were transferred to a 15-ml tube (BD Biosciences), collected at the bottom of the tube by gravity, resuspended in 1–2 ml of fresh medium, and seeded into an uncoated, 6-cm bacterial Petri dish (SPL Life Sciences). Approximately 200 colony fragments were seeded on a 6-cm bacterial Petri dish. The colony fragments were cultured in suspension for 4 days in the EB medium (DMEM/F12, 10 % Knockout SR, 1 % NEAA, 0.1 mM β-mercapto ethanol, and 1 % P/S; all from Invitrogen) and were supplemented with dorsomorphin (5 μM) (Sigma-Aldrich) and SB431542 (10 μM) (Tocris, Bristol, UK).

Fifty to sixty EBs were transferred to a 3-cm Matrigel-coated dish and were cultured for 6 days in neural progenitor (NP) selection medium (DMEM/F12, 1 mM L-Glutamine, 1 % NEAA, 0.1 mM β-mercapto ethanol, 0.5 % N2 supplement, and 20 ng/ml basic fibroblast growth factor (bFGF); CHA Meditech, Seoul, Korea) with medium change every 2 days.

Neural rosettes were mechanically dissected with pulled glass pipette (World Precision Instruments, Sarasota, FL, USA) and cultured in 6-cm Matrigel-coated dishes containing NP expansion medium (DMEM/F12, 1 mM L-Glutamine, 1 % NEAA, 0.1 mM β-mercapto ethanol, 0.5 % N2 supplement, 2 % B27 supplement, and 20 ng/ml bFGF). When the transferred rosettes covered approximately 90 % of the dishes, they were treated with Accutase (Invitrogen) for passaging.

### Neuronal differentiation

For neuronal differentiation, the iPSC-neural precursor cells (iPSC-NPCs) were plated at 2×10^4^ cells/cm^2^ on a 12-mm, round, poly-L-ornithine (20 μg/ml)/laminin (10 μg/ml) (all from Sigma-Aldrich)-coated glass cover slip. Cells were maintained in neurobasal medium containing 1 % P/S, 1×Glutamax, 1 % N2, 2 % B27 supplement, 1 % FBS, brain-derived neurotrophic factor (BDNF) (10 ng/ml) (Peprotech, Rocky Hill, NJ, USA), and glial cell-derived neurotrophic factor (GDNF) (10 ng/ml) (Peprotech) for 21 days. Half of the medium was replaced every two days.

### Whole-cell patch clamp

The cover slip with cultured cells was transferred to the recording chamber (Warner Instruments, Hamden, CT, USA) and placed on the microscope (Olympus, Tokyo, Japan) while continuously perfused with cerebrospinal fluid containing: 124 mM NaCl, 3 mM KCl, 1.3 mM MgSO_4_, 1.25 mM NaH_2_PO_4_, 26 mM NaHCO_3_, 2.4 mM CaCl_2_-2H_2_O, and 10 mM glucose (all from Sigma-Aldrich). The solution was continuously aerated by O_2_ 95 %/CO_2_ 5 % mixed gas at RT.

A glass capillary pipette approached the cell surface. After that, negative pressure was provided to let the pipette go giga seal and whole-cell mode. Internal pipette solution contained 115 mM K-gluconate, 10 mM KCl, 10 mM HEPES, 10 mM EGTA, 5 mM Mg-ATP, and 0.5 mM Na^2+^-GTP (all from Sigma-Aldrich), with pH 7.3 and 280–285 mOsm. Holding potential was −60mV. Na^+^ current was recorded in voltage clamp mode, and electrical stimulation was given with a range from −60 mV to +50 mV (+10 mV per each step). After that, cells went to current clamp mode for testing action potential generation. Cells received 15 steps of current (initial level = 0 pA, Δ3–10 pA per step) according to their membrane capacity. To verify that currents and spikes were mediated by Na^+^ channels, tetrodotoxin (TTX) (0.5 μM) (Sigma-Aldrich) was added to the bath for 5–10 min, and currents and spikes were retested.

### Spinal cord injury and cell transplantation

All experiments were performed according to international guidelines on the ethical use of animals, and the number of animals used was minimized. All protocols were approved by the Animal Care and Use Committee of Yonsei University College of Medicine. Adult male Imprinting Control Region (ICR) mice (35–40 g) (Orient Bio, Gyeonggido, Korea) were used for the SCI model. Before surgery, Cefazoline (20 mg/kg) (Yu-han, Seoul, Korea) was injected into the animals. For anesthesia, Zoletil 50 (30 mg/kg) (Virbac, Carros, France) and Rompun (10 mg/kg) (Bayer Korea, Seoul, Korea) were injected. After anesthesia, laminectomy was performed at the 11th thoracic level. Compression injury (for 10 seconds) was carried out using 0.2-mm spacer self-closing forceps at the 11th thoracic level. Cell transplantation was performed 9 days after SCI. Animals were divided into two groups: group 1 (1×PBS injection) and group 2 (diPSC-derived NPCs, 5×10^5^ cell injection). Two microliters of cell suspension containing 5×10^5^ cells was injected into the injury epicenter by using a micro injector (KDS Scientific, Holliston, MA, USA) attached to a pulled glass capillary needle (World Precision Instruments) (inner diameter of 150–200 μm) at a rate of 1 μl/min. For immune suppression, Cyclosporine A (10 mg/kg) (Chong Kun Dang, Seoul, Korea) was administered to all animals until sacrifice.

### Behavioral tests

To confirm whether cell transplantation improved hindlimb functional recovery, the open-field locomotor test was performed every week for 6 weeks by using Basso Mouse Scale (BMS) scoring, and footprint analysis was performed only at the final week. Briefly, both hindlimbs were soaked in paint, and the animals walked on drawing paper. Stride length, stance length, and sway length were measured.

### Immunofluorescence staining

Five weeks after cell transplantation, animals were sacrificed by heart perfusion with saline and 4 % PFA (Merck, Darmstadt, Germany)/1×PBS. Spinal cord tissue was obtained and incubated in 4 % PFA for 24 h at 4 °C. After fixation, samples were incubated in 30 % sucrose (Sigma-Aldrich)/1×PBS solution at 4 °C. Tissues were embedded into optimal cutting temperature compound (Cellpath, Hemel, Hempstead, UK), incubated at −70 °C, and cut into slices of 20-μm thickness. Immunofluorescence staining was performed as follows. Samples were washed with ice-cold 1×PBS, followed by blocking with 10 % normal donkey serum (Jackson ImmunoResearch)/1×PBS containing 0.3 % Triton X-100 (Sigma-Aldrich) for 1 h at RT. Primary antibodies (Additional file 1) were treated either for 1 h at RT or overnight at 4 °C. After three washes with ice-cold 1×PBS, fluorescent dye-conjugated secondary antibodies (Jackson ImmunoResearch) were treated for 1 h at RT. The samples were mounted with a mounting solution (Vector Laboratories) and analyzed by using an Olympus BX51 fluorescence microscope (Olympus) and LSM 700 confocal microscope (Zeiss, Oberkochen, Germany). The percentages of Nestin-, beta-III tubulin (TUJ1)-, microtubule-associated protein 2 (MAP2)-, glial fibrillary acidic protein (GFAP)-, Neuron/glia-type 2 (NG2)-, and Olig2-positive cells among total human nuclei (HNU)-positive cells within 0.3 mm^2^ were examined (three independent regions).

### Hematoxylin-and-eosin staining

To visualize the injured spinal cord, each longitudinal section of the spinal cord encompassing the injury site was stained with hematoxylin and eosin (H&E). For analysis of longitudinal section, midsagittal sections of spinal cord were used. The pixel density within 2×4 mm^2^ at the injury site was quantified by using NIH Image J software (National Institutes of Health, Bethesda, MD, USA). For analysis of axial section, the pixel density at the lesion epicenter and 0.8 and 0.4 mm rostral and caudal to the epicenter, respectively, was measured by using NIH Image J software. For H&E staining, slides were placed into hematoxylin for 5 min and rinsed for 3 min. The pink hematoxylin stain was converted to blue by adding Scott’s tap water until the sections turned blue. The slides were rinsed before being stained in eosin (1 % (wt/vol)) for 15 seconds with a subsequent wash for 3 min. The sections were then dehydrated in two washes of absolute alcohol and in two washes of xylene for 5 min each before being mounted and covered with glass cover slips for observation.

### Statistical analysis

Student’s *t* tests were performed to assess differences between the two groups. One-way analysis-of-variance tests were performed to assess difference among three groups with the Student-Newman-Keuls test. The data were presented as the mean ± standard error of the mean. *P* values of less than 0.05 were considered statistically significant. All data were analyzed by using MedCalc statistical software (MedCalc Software, Ostend, Belgium).

## Results

### Isolation of disc cells for induced pluripotent stem cell generation from a patient with spinal cord injury

**Fig. 1:**
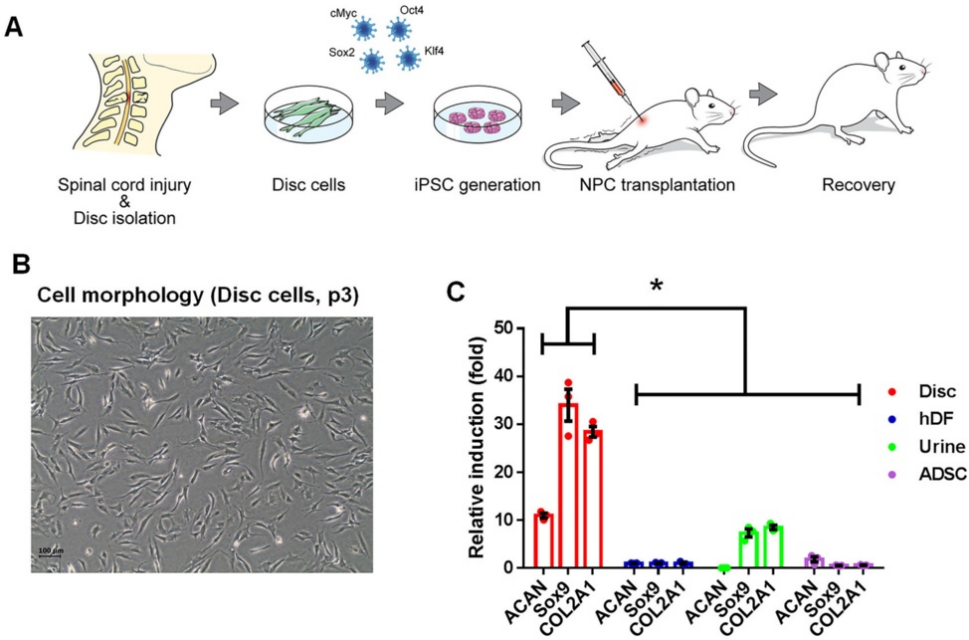
Experimental design and characteristics of human intervertebral disc-derived cells. **a** Schematic illustration of the experimental paradigm adopted in this study. **b** Morphology of the cells (passage 3) derived from human intervertebral discs. **c** Quantitative real-time reverse transcription-polymerase chain reaction demonstrated high expression of chondrogenic marker genes, aggrecan (*ACAN*), SRY (sex determining region Y)-box9 (*Sox9*), and type II collagen (*Col2A1*), in the disc cells compared with adult dermal fibroblasts, urine cells, and adipose-derived stromal cells (ADSCs). All cell types were analyzed at passage 3. *P < 0.01 (*n* = 3). *hDF* human dermal fibroblast, *iPSC* induced pluripotent stem cell, *NPC* neural precursor cell

In this study, we examined whether iPSCs could be generated from typically discarded disc cells isolated from surgically removed human intervertebral discs and whether NPCs differentiated from the diPSCs reversed the locomotor dysfunction of an animal SCI model (Fig. 1a). A large number of the disc-originated cells in our culture displayed the morphology of nucleus pulposus (NP) cells, which are less elongated and more branched than annulus fibrosus (AF) cells (Fig. 1b) [13]. Consistent with this morphological observation, NP marker genes such as aggrecan (*ACAN*), SRY (sex determining region Y)-box 9 (*Sox9*), and type II collagen (*Col2A1*) were abundantly expressed in the disc cell population (Fig. 1c) [14].

### Generation and characterization of disc cell-derived induced pluripotent stem cells from the cultured disc cells

**Fig. 2:**
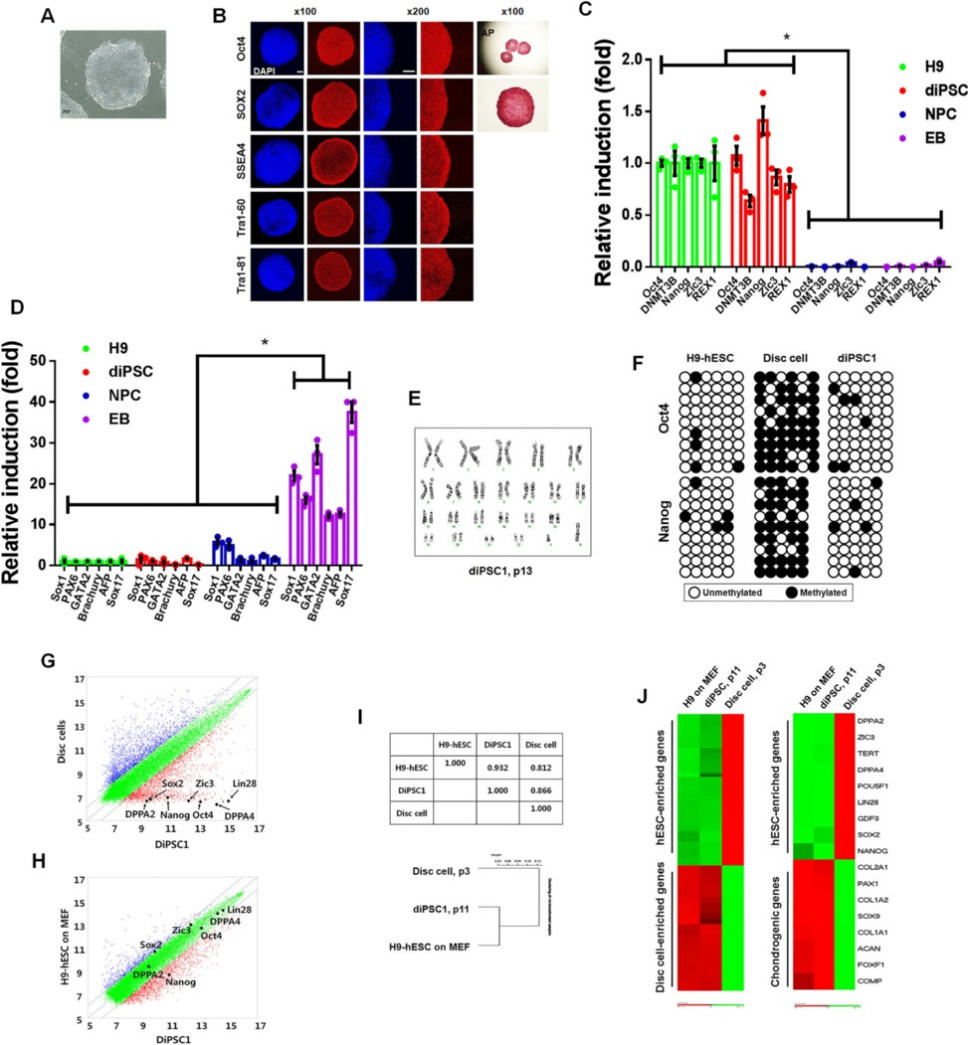
Characterization of diPSCs. **a** A phase-contrast image of diPSC colonies, which were generated and cultured for 15 passages. **b** diPSC colonies at passage 15 were immunostained with representative pluripotency markers (Tra1-81, Tra1-60, SSEA4, Sox2, and Oct4) and were also stained for alkaline phosphatase. **c** Quantitative real-time RT-PCR was performed to detect the levels of multiple undifferentiated cell markers, such as Oct4, Nanog, DNMT3B, Zic3, and Rex1. *P < 0.01 (*n* = 3). **d** The expression levels of the representative marker genes for the ectoderm (*Sox1*, *Pax6*), mesoderm (*GATA2*, *Brachyury*), and endoderm (*AFP*, *Sox17*) lineages were measured by quantitative real-time RT-PCR. **e** G-banding analysis of diPSC1 at passage 13. **f** Bisulfite sequencing analysis to examine the methylation pattern of the *Oct4* and *Nanog* promoters in H9-hESCs, disc cells, and diPSC. **g**, **h** The scatter plots compare gene expression levels between disc cells and diPSC1 (g) and between H9-hESCs and diPSC1 (h). **i** Pair-wise Pearson’s correlation coefficients among gene expression profile data for H9-hESCs, diPSC1, and disc cells are shown (*top*). Hierarchical clustering of the global expression profiles of H9-hESCs, diPSC1, and disc cells is shown (*bottom*). **j** A heatmap of the expression of the disc cell and hESC-enriched genes from H9-hESCs, diPSCs (p11), and disc cells (p3) (*left*). The list of disc cell and hESC-enriched genes is shown in Additional file 4. In addition, a heatmap of the expression of chondrogenic and hESC-enriched genes is shown on the right. The list of disc cell and hESC-enriched genes is shown in Additional file 5. The genes shown in green represent upregulation of expression, whereas the genes in red represent downregulation. *diPSC* disc cell-derived induced pluripotent stem cell, *EB* embryoid body, *hESC* human embryonic stem cell, *NPC* neural precursor cell, *RT-PCR* reverse transcription-polymerase chain reaction

hPSC-like colonies appeared approximately 18–20 days after the disc cells were transduced with a mixture of retroviral vectors expressing each of *Oct4*, *Sox2*, *Klf4*, and *c-Myc* genes (Fig. 2a). The efficiency of iPSC formation from disc cells was approximately 0.1 %. A DNA fingerprinting analysis confirmed that the diPSCs were derived from the original disc cells that were used for the iPSC generation (Additional file 3). Both immunochemistry and real-time RT-PCR experiments demonstrated that the iPSC lines robustly expressed undifferentiated cell markers Oct4, Sox2, SSEA4, Tra1-60, Tra1-80, Nanog, DNMT3B, Zic3, and Rex1 (Fig. 2b, c). On the other hand, low-level expression of representative markers for neuroectoderm (Sox1 and Pax6), mesoderm (Gata2 and Brachyury), and endoderm (AFP and Sox17) was detected (Fig. 2d). G-banding analysis indicated that there were no gross chromosomal aberrations in the diPSCs (Fig. 2e). Consistent with the high level of *Oct4* and *Nanog* gene expression, the regulatory regions of the two genes were hypomethylated in both diPSCs and hESCs but not in their original disc cells (Fig. 2f). As previously reported in many other laboratories, PCR analysis showed that the exogenously delivered *Oct4*, *Sox2*, *Klf4*, and *c-Myc* transgenes were turned off in diPSCs but that their endogenous counterparts were actively expressed (data not shown). Next, we closely examined global gene expression patterns of diPSCs, H9-hESCs, and disc cells. Scatter plot, correlation matrix, hierarchical clustering, and heatmap analyses all demonstrated that diPSCs had similar gene expression patterns to hESCs but not to their original disc cells (Fig. 2g-j). (Additional information is shown in more detail in Additional files 4 and 5.) All together, these results indicated that the diPSC colonies formed in our study displayed characteristics similar to hESCs.

### Pluripotency of disc cell-derived induced pluripotent stem cells

**Fig. 3:**
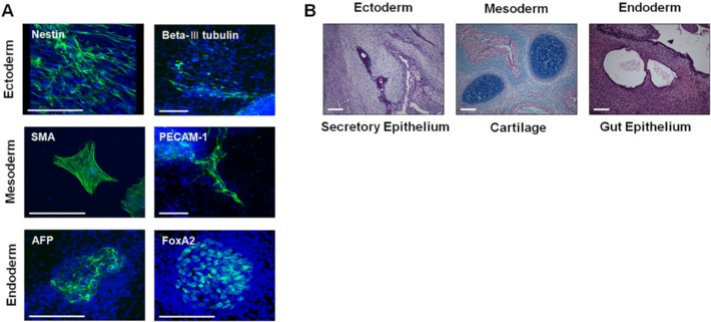
The differentiation of diPSCs into derivatives of the three germ layers. **a** diPSCs (p15) were spontaneously differentiated in vitro, and the expression of representative markers of ectoderm (Nestin and beta-III tubulin), mesoderm (SMA and PECAM-1), and endoderm (AFP and FoxA2) was examined. **b** The derivatives of the three germ layers were detected in teratomas approximately 9–10 weeks after administration of diPSCs (2×10^6^ cells) into nonobese diabetic/severe combined immunodeficiency mice. *AFP* alpha-fetoprotein, *diPSC* disc cell-derived induced pluripotent stem cell, *FoxA2* forkhead box A2, *PECAM-1* platelet endothelial cell adhesion molecule-1, *SMA* smooth muscle actin

In spontaneous differentiation conditions, diPSCs were shown to differentiate into derivatives of all three germ layers, as detected by immunostaining of the following representative germ layer markers: Nestin and beta-III tubulin for ectoderm, smooth muscle actin (SMA) and platelet endothelial cell adhesion molecule-1 (PECAM-1) for mesoderm, and alpha-fetoprotein (AFP) and forkhead box A2 (FoxA2) for endoderm (Fig. 3a). Furthermore, when injected into NOD/SCID mice, diPSCs formed complex teratomas consisting of tissues derived from all three germ layers (Fig. 3b).

Taken together, both in vitro and in vivo results demonstrated that the diPSCs retained the capability of pluripotency.

### Differentiation of disc cell-derived induced pluripotent stem cells into neural precursor cells

**Fig. 4:**
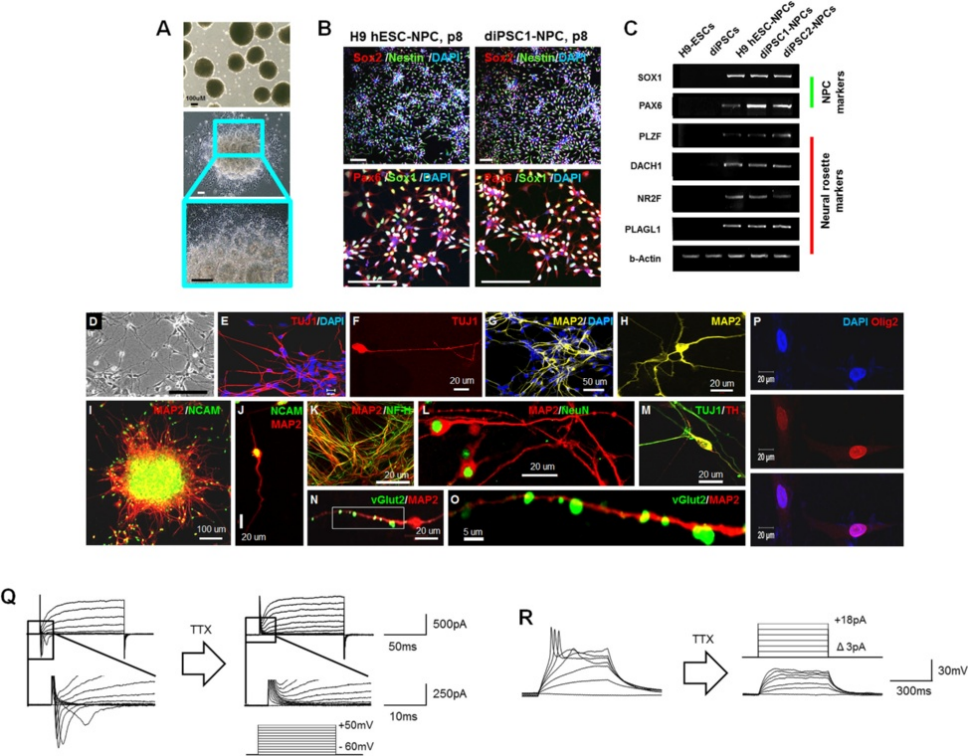
Generation and characterization of diPSC-NPCs. **a** diPSCs were coaxed into NPCs (diPSC-NPCs) via EB formation in the presence of dorsomorphin (DM) and SB431542 (SB). EBs (*top panel*) and neural rosettes (*middle and bottom panels*) are shown. **b** The NPCs selected and expanded from neural rosettes were immunostained with neural precursor markers Sox2, Nestin, Sox1, and Pax6. *Bottom* panels are higher-magnification images of *top* panels. **c** RT-PCR analysis showed the expression of markers for both NPCs (Sox1 and Pax6) and neural rosettes (PLZF, DACH1, NR2F, and PLAGL1) in NPCs derived from hESCs, diPSC1, and diPSC2. **d** A differential interference contrast image of neurons differentiated from diPSC-NPCs. **e**, **f** TUJ1^+^ neurons at low and high magnifications, respectively. **g**, **h** MAP2^+^ neurons at low and high magnifications, respectively. (**i** and **j**) NCAM^+^ neurons at low and high magnifications, respectively. **k** Dense NF^+^/MAP2^+^ axon fibers. **l** MAP2^+^/NeuN^+^ neurons. **m** TUJ1^+^/TH^+^ dopaminergic neuron at 21 days of differentiation. **n**, **o** vGlut2^+^/MAP2^+^ glutamatergic neuron at 21 days of differentiation. The image in (**o**) was a high magnification of the white box in (**n**). **p** Some diPSC-NPCs differentiated into Olig2-positive oligodendrocytes. **q** Na^+^ channel-mediated current was examined to prove the functionality of the differentiated neurons. Na^+^ currents were produced in neurons. Voltage steps from −60 mV to +50 mV were applied to the patched cells, and several positive currents were found (*left*, *n* = 3 cells/total 15 cells). By applying 0.5 μM TTX, a Na^+^ channel blocker, currents were totally abolished (*right*). Boxes were amplified to show Na^+^ currents and their disappearance precisely. **r** Action potentials were also generated in differentiated neurons. Narrow spikes were recorded in the cells which showed Na^+^ currents (*left*). Protocol involved sequential steps in stimulation (*right, top*). Detected action potential spikes were also blocked by TTX, just as Na^+^ current in (**q**) (*right, bottom*). *diPSC* disc cell-derived induced pluripotent stem cell, *EB* embryoid body, *hESC* human embryonic stem cell, *MAP2* microtubule-associated protein 2, *NCAM* neural cell adhesion molecule, *NF* neurofilament, *NPC* neural precursor cell, *Olig2* oligodendrocyte lineage transcription factor 2, *RT-PCR* reverse transcription-polymerase chain reaction, *Sox2* SRY (sex determining region)-box 2, *TH* tyrosine hydroxylase, *TTX* tetrodotoxin, *TUJ1* beta-III tubulin, *vGlut2* vesicular glutamate transporter 2.

diPSCs were differentiated into NPCs via the previously described EB-based method with slight modifications (Fig. 4a) [15]. Neural rosettes were mechanically selected and seeded as a single cell in the neural expansion medium to culture the NPCs. The NPCs were passaged eight times in vitro and robustly expressed NPC makers, such as Nestin, Sox2, Pax6, and Sox1 (Fig. 4b). Semi-quantitative RT-PCR also showed the expression of both NPC and neural rosette markers in hPSC-derived NPCs (H9-NPCs, diPSC1-NPCs, and diPSC2-NPCs) but not in H9-hESCs and diPSCs (Fig. 4c).

### Neuronal differentiation in vitro

Next, we investigated whether the diPSC-NPCs could be further coaxed into neurons in vitro. The neurons generated by differentiation of diPSC-NPCs displayed typical neuronal morphology with small soma and long processes (Fig. 4d). Twenty-one days after differentiation, most of the neurons were expressing beta-III tubulin (TUJ1^+^) (Fig. 4e, f), microtubule-associated protein 2 (MAP2) (Fig. 4g, h), neural cell adhesion molecule (NCAM) (Fig. 4i, j), neurofilament heavy (NF-H) (Fig. 4k), and neuronal nuclei (NeuN) (Fig. 4l). Among them, tyrosine hydroxylase (TH)^+^ dopaminergic neurons (Fig. 4m) and vesicular glutamate transporter 2 (vGlut2)^+^ glutamatergic neurons (Fig. 4n, o) were also detected. In addition, diPSC-NPCs also differentiated into Olig2^+^oligodendrocytes in serum (1 % FBS (vol/vol))-containing medium (Fig. 4p).

The electrophysiological properties of the diPSC-derived neurons were examined by whole-cell patch clamp recordings. In voltage clamp mode, Na^+^ currents were found in differentiated neurons (Fig. 4q). Cells were held at −60 mV, and several current peaks were recorded when sequential stimulations (from −60 mV to +50 mV) were applied. The maximum peak amplitude was −556.6 pA. For confirmation of excitability, another piece of evidence indicating normal neuronal function, we used evoked action potentials in the current clamp mode. Action potential spikes resulted (Fig. 4r), but they appeared only in the cells that produced Na^+^ currents. This result implied that the action potentials we detected were caused by the same Na^+^ channels that elicited positive current in Fig. 4q.

To confirm that the channels that produced currents and spikes were Na^+^ channels, Na^+^ channel antagonist TTX was added in the bath for 5–10 min. TTX blocked Na^+^ channels completely, and Na^+^ currents and action potential spikes totally disappeared (Fig. 4q and r, *right panel*), supporting the assertion that the currents and spikes were mediated by Na^+^ channels. diPSC-derived neurons expressed a certain level of Na^+^ channels and generated action potentials, indicating that the diPSC-derived NPCs had differentiated into mature neurons.

### Functional and structural recovery in a mouse model of spinal cord injury

**Fig. 5:**
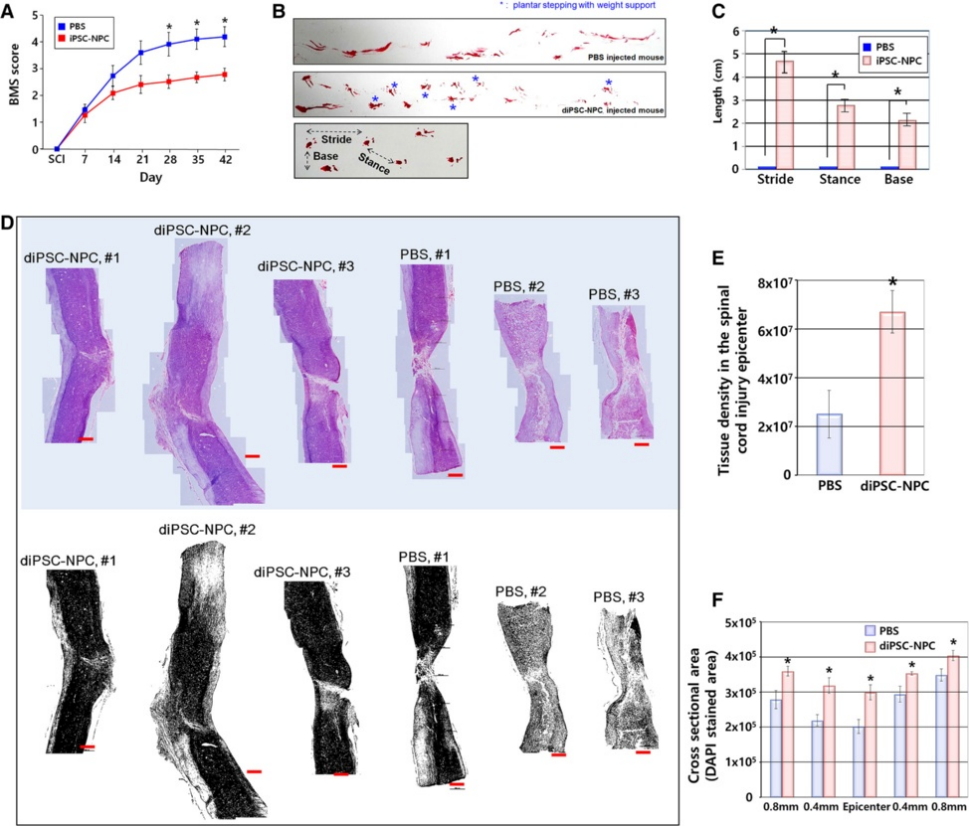
Transplantation of diPSC-NPCs enhanced the functional recovery and prevented the atrophy caused by spinal cord injury. **a** Basso Mouse Scale (BMS) scores showed a significant functional recovery of hindlimbs in the diPSC-NPC-transplanted group. **b** Footprint analysis showed that the diPSC-NPC-transplanted mice displayed better walking performance (plantar stepping, blue asterisk) than the control group. To obtain statistically reliable data, we strictly followed the standard protocol of each behavioral test and tried to include many animals per group (PBS group: *n* = 14, diPSC-NPC group: *n* = 20). **c** The lengths of base, stride, and stance were also significantly increased in the diPSC-NPC-transplanted group compared with the control group. **d** Longitudinal sections of the spinal cord isolated from PBS (control)- or diPSC-NPC-injected mice were stained with hematoxylin and eosin. **e** Tissue density of the spinal cord near the injury epicenter was measured by using National Institutes of Health Image J software. **f** The cross-sectional area of the spinal cord spanning 1.6 mm in the anterior-posterior axis with the injury epicenter in the middle was examined for tissue density. *P < 0.05*.* Data are presented as the mean ± standard error of the mean. *diPSC* disc cell-derived induced pluripotent stem cell, *iPSC* induced pluripotent stem cell, *NPC* neural precursor cell, *PBS* phosphate-buffered saline, *SCI* spinal cord injury

To investigate whether transplantation of diPSC-NPCs enhanced functional recovery of the hindlimbs of mice with SCI, both the open-field locomotor test (every week) and footprint analysis (final week) were performed. In the open-field locomotor test, diPSC-NPC transplantation significantly reversed hindlimb dysfunction compared with the control (PBS-injected) group. The transplanted mice frequently showed plantar stepping with weight support (Fig. 5a). In footprint analysis, diPSC-NPC-transplanted mice had greatly improved walking performance on plantar stepping compared with PBS-injected mice, as judged by the significant increase in the base, stance, and stride lengths (Fig. 5b, c). These results indicated that transplantation of the diPSC-NPCs ameliorated the hindlimb dysfunction caused by SCI.

Next, we investigated whether transplantation of diPSC-NPCs protected the spinal cord from damage in the SCI mouse model. The H&E staining results showed that significantly less damage of the spinal cord tissue was detected in diPSC-NPC-transplanted mice than PBS-injected control mice (Fig. 5d). Tissue sparing at each level of the spinal cord encompassing the injury epicenter was significantly greater in diPSC-NPC-transplanted mice than control mice (Fig. 5e, f), suggesting that the transplanted diPSC-NPCs played a beneficial role in structural recovery from SCI.

### Differentiation of disc cell-derived induced pluripotent stem cell-derived neural precursor cells in vivo

**Fig. 6:**
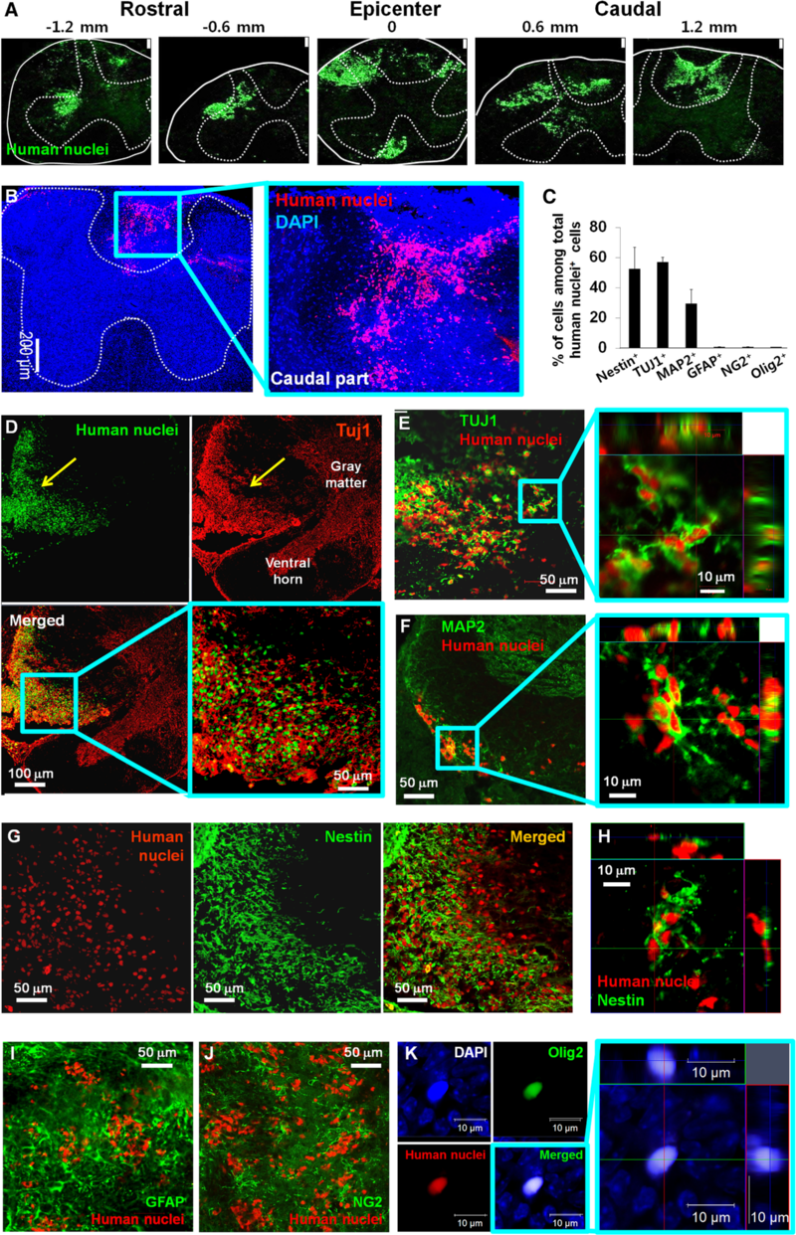
Immunohistological analysis at 5 weeks after transplantation of diPSC-NPCs into spinal cord-injured mice. **a**, **b** Images of horizontal sections of the spinal cord were taken. **c** The percentage of each neural cell type derived from transplanted diPSC-NPCs among total human nuclei^+^ cells is indicated in the graph. **d**, **e** Double immunostaining was performed with antibodies recognizing human nuclei and Tuj1. **f** A horizontal section of the spinal cord was immunostained with antibodies for human nuclei and MAP2. **g** Double immunostaining of a horizontal section of the spinal cord was performed with antibodies for human nuclei and Nestin. **h** Z-axis scanning of human nuclei and Nestin double-positive cells. **i** Double immunostaining with antibodies specific for human nuclei and GFAP was performed. **j** Double immunostaining with antibodies specific for human nuclei and NG2 was performed. **k** An example of human nuclei^+^ cell that was co-stained with Olig2 is shown. Data are presented as the mean ± standard error of the mean. *diPSC* disc cell-derived induced pluripotent stem cell, *GFAP* glial fibrillary-associated protein, *MAP2* microtubule-associated protein 2, *NG2* neural/glial anigen-2, *NPC* neural precursor cell, *Olig2* oligodendrocyte lineage transcription factor 2, *TUJ1* beta-III tubulin

Five weeks after transplantation, we examined whether the transplanted diPSC-NPCs differentiated into various neural cell types. First, we examine the distribution of the cells originated from the transplanted diPSC-NPCs by using HNU antibody. A series of transverse sections showed that the transplanted human cells were existing from the epicenter to both rostral and caudal areas of the spinal cord and these cells survived at least 5 weeks after transplantation (Fig. 6a). The HNU^+^ cells were detected across distances at least 2.4 mm along the spinal cord with the injection site in the middle, indicating that the transplanted diPSC-NPCs migrated actively from the injection site (Fig. 6a). The migrated cells integrated into the spinal cord without preference for white or gray matter (Fig. 6a, b, d).

Next, we performed double immunostaining with antibodies recognizing HNU and Tuj1 to examine the percentage of neurons (HNU^+^ and Tuj1^+^) differentiated from the transplanted diPSC-NPCs among total HNU^+^ cells. Our result showed that a significant portion of the HNU^+^ cells (~57 %) were also stained with Tuj1 antibody (Fig. 6c, d, e). In addition, we found that many HNU^+^ cells (~29.5 %) were also positive for MAP2, a marker for mature neurons (Fig. 6c, f). We also carried out double immunostaining for HNU and Nestin antigens to measure the percentage of diPSC-NPCs which were still remaining undifferentiated at 5 weeks after transplantation. Our result showed that approximately 52.6 % of HNU^+^ cells were also positive for Nestin (Fig. 6c, g, h). In contrast, differentiation of the transplanted diPSC-NPCs into astrocytes (Fig. 6c, i), oligodendrocyte progenitor cells (Fig. 6c, j), and oligodendrocytes (Fig. 6c, k) occurred rarely because only a few HNU and GFAP double-positive, HNU and NG2 double-positive, and HNU and Olig2 double-positive cells were detected, respectively. Taken together, our results demonstrated that transplanted diPSC-NPCs preferentially differentiated into neurons in vivo, which is at least partly responsible for the functional recovery observed after transplantation in a mouse model of SCI.

## Discussion

To date, a variety of somatic cell types have been used to generate human iPSCs: fibroblasts [9, 16–20], circulating T cells [9, 10, 20], cord blood stem cells [18, 21], neural stem cells [22], molar mesenchymal stromal cells [23], aortic smooth muscle cells [24], keratinocytes [6], and melanocytes [25]. Intriguingly, certain populations of somatic cells have their own unique characteristics with regard to reprogramming capability, resulting in variable experimental schedules and outcomes in iPSC generation. For example, only Oct4 was required to reprogram neural stem cells into iPSCs [22], and ketatinocytes were approximately 100-fold more efficient at iPSC generation than fibroblasts [6].

In most cases, patient-specific iPSCs were generated by using fibroblasts obtained from the patient by a punch skin biopsy because of convenience. However, this biopsy involves a painful surgical procedure and requires a significant amount of time to expand the cell population sufficiently for iPSC generation. Therefore, it is of great use to take advantage of “to-be-discarded” tissues obtained from a surgical procedure as a source of somatic cells for iPSC-based, patient-specific cell therapy. Our study proposed a paradigm of iPSC generation using waste tissue from emergency surgery on a patient with SCI and these iPSCs could be a potential therapeutic autologous stem cells for SCI.

Human intervertebral disc cells are difficult to obtain in normal situations, and there have been no reports describing the use of disc-derived cells for iPSC generation. However, during emergency surgery on patients with SCI, this disc tissue is often removed as to-be-discarded “waste” and could be a useful source for iPSC-based autologous cell replacement therapy.

We showed that disc cells derived from the intervertebral disc removed from a patient with SCI abundantly expressed disc cell marker genes such as *aggrecan*, *Sox9*, and *Col2A1*. Genome-wide gene expression profile analysis demonstrated a robust expression of chondrocyte genes, further supporting that the cells were derived from the intervertebral disc (Fig. 2j) (Additional file 4). The disc cells were efficiently reprogrammed to iPSCs (named “diPSCs”) that successfully met all the criteria of pluripotent stem cells. Therefore, diPSCs originating from the intervertebral disc of a patient with SCI will provide a useful therapeutic paradigm for the treatment of SCI in the future.

The diPSCs were readily converted to NPCs by a modified EB-mediated neural differentiation method with dual-smad inhibition [26]. Neural rosette formation and expression of neural markers were comparable between the NPCs derived from H9-hESCs and from diPSCs. In poly-l-ornithine/laminin-coated dishes with neurobasal/B27-based medium, the diPSC-NPCs were efficiently differentiated into neurons. We detected some glutamatergic neurons expressing vGlut2 and also TH^+^ dopaminergic neurons (Fig. 4d). On the contrary, serotonergic neurons and GABAergic neurons were hardly detected (data not shown). To examine the functionality of the neurons derived from diPSC-NPCs, we performed whole-cell patch clamp at 3 weeks after differentiation of NPCs. Our results showed that the differentiated neurons have enough voltage-gated Na^+^ channels to mediate the generation of action potentials and Na^+^ currents. This result is consistent with previous studies that also recorded Na^+^ currents and action potentials to identify NPC differentiation level [27, 28].

When transplanted into the spinal cord of an SCI mouse model, a large number of engrafted NPCs were still alive at 5 weeks after transplantation. Intriguingly, a significant portion of the HNU^+^ cells were found to be Nestin^+^ NPCs (~52.6 %), TUJ1^+^ neurons (~57 %), and MAP2^+^ neurons (~29.5 %), whereas only a small number of the engrafted diPSC-NPCs differentiated into astrocytes (GFAP^+^), oligodendrocyte progenitor cells (NG2^+^), and oligodendrocytes (Olig2^+^). These results are similar to those of another group, who reported that the engraftment of hiPSC-derived neurospheres produced Nestin^+^ NPCs (~10.7 %), TUJ1^+^ neurons (~49.1 %), and APC^+^ oligodendrocytes (~3 %) [4]. However, this group detected approximately 17 % GFAP^+^ astrocytes, which is significantly higher than what we observed. We speculate that this discrepancy might be caused by differences in the state of engrafted cells (NPCs versus neurospheres), experimental schedule (analyzed at 35 days versus 47 days after transplantation), and the environment surrounding the engrafted cells. In fact, a significant discrepancy has been reported in differentiation capability between NPCs transplanted into the lesion epicenter and NPCs engrafted onto the edge of injury epicenter [29], and the percentage of neural cell types has been shown to differ depending on length of time to analysis after transplantation [4].

Interestingly, neurologic improvement was evident despite the low capability of differentiation into oligodendrocytes in our study and in reports from other groups [4, 30]. Because remyelination of axons by oligodendrocytes is one of the essential processes required for behavioral recovery after SCI, it is tempting to speculate that secreted factor(s) from the engrafted NPCs or their differentiated derivatives may induce endogenous oligodendrocyte-mediated remyelination.

Although tumorigenic potential has been regarded as the most serious issue in ESC and iPSC transplantation research, no tumor formation was observed in this study. This observation is consistent with the absence of Oct4^+^ cells in the diPSC1-NPC culture at passage 8 (data not shown), suggesting that diPSCs were all differentiated by the neural differentiation protocol used in this study.

Our study demonstrated that surgically removed, to-be-discarded “waste” tissue can be reused to form patient-specific iPSCs. Cells obtained from an emergency surgery, especially those that are difficult to access in normal situations, are a valuable resource for studying cell-specific reprogramming processes, eventually deepening our understanding of the cellular reprogramming process. Furthermore, diPSCs may be used for autologous cell replacement therapy to treat patients with SCI.

In this study, iPSCs were generated from human intervertebral disc tissue removed surgically to stabilize the spine after SCI. We confirmed that chondrogenic intervertebral disc cells were completely changed to pluripotent stem cells. The characteristics of the diPSCs were similar to those of hESCs but were different from the original disc cells. diPSC-NPCs were efficiently coaxed into mature neurons with the typical action potential of neurons. We confirmed in vivo that transplantation of diPSC-NPCs improved the functional recovery of spinal cord-injured mice and prevented spinal cord atrophy. A large number of transplanted diPSC-NPCs either remained Nestin^+^ NPCs or differentiated into TUJ1^+^ or MAP2^+^ neurons or both. No tumor formation was observed in our study.

## Conclusions

In this study, we demonstrated that the “to-be-discarded” intervetebral discs removed during spinal operation could be a rare and valuable biological source for generating iPSC, which would be of great use to study epigenetic memory in cellular reprogramming, and also provide an autologous cell source for future cell replacement therapy.

## Additional files

Additional file 1: Table S5. List of the antibodies used in this study. Additional file 2: Table S4. List of primer pairs used in this study. Additional file 3: Table S1. DNA fingerprint analysis. Additional file 4: Table S2. Lists of human embryonic stem cell (hESC)-enriched genes and disc cell-enriched genes shown in Fig. 2j (*left*). Additional file 5: Table S3. Lists of human embryonic stem cell (hESC)-enriched genes and chondrogenic genes shown in Fig. 2j (*right*).

## Abbreviations

AFP: Alpha-fetoprotein
bFGF: Basic fibroblast growth factor
diPSC: Disc cell-derived induced pluripotent stem cell
DMEM: Dulbecco’s modified Eagle’s medium
EB: Embryoid body
ESC: Embryonic stem cell
FBS: Fetal bovine serum
FITC: Fluorescein isothiocyanate
GFAP: Glial fibrillary-associated protein
H&E: Hematoxylin and eosin
hESC: Human embryonic stem cell
HNU: Human nuclei
hPSC: Human pluripotent stem cell
iPSC: Induced pluripotent stem cell
Klf4: Kruppel-like factor 4
MAP2: Microtubule-associated protein 2
NEAA: Non-essential amino acid
NG2: Neural/glial anigen-2
NIH: National Institutes of Health
NOD/SCID: Nonobese diabetic/severe combined immunodeficiency
NP: Nucleus pulposus
NPC: Neural precursor cell
Oct4: Octamer-binding transcription factor 4
Olig2: Oligodendrocyte lineage transcription factor 2
P/S: Penicillin/streptomycin
PBS: Phosphate-buffered saline
PCR: Polymerase chain reaction
PFA: Paraformaldehyde
qRT-PCR: Quantitative reverse transcription-polymerase chain reaction
RT: Room temperature
RT-PCR: Reverse transcription-polymerase chain reaction
SCI: Spinal cord injury
Sox2: SRY (sex determining region)-box 2
SRY: Sex determining region
TH: Tyrosine hydroxylase
TTX: Tetrodotoxin
TUJ1: beta-III tubulin
vGlut2: Vesicular glutamate transporter 2
