# Supplementary material for: Human-induced pluripotent stem cells generated from intervertebral disc cells improve neurologic functions in spinal cord injury
Source: Stem Cell Res Ther. 2015 Jun 24;6(1):125. doi: 10.1186/s13287-015-0118-x (PMC4529688; doi:10.1186/s13287-015-0118-x)
Supplement: Additional file 1: Table S5. — List of the antibodies used in this study. [file 13287_2015_118_MOESM1_ESM.pdf]

Table 5. List of the antibodies used in this study

Antibody (Host)	Company	Cat. No	Dilution factor
#TUJ1 (Chicken)	Millipore	AB9354	1:1000
#TUJ1 (Rabbit)	Abcam	AB18207	1:1000
#MAP2 (Mouse)	Abcam	AB11267	1:500
#NCAM (Rabbit)	Abcam	AB75813	1:200
#NF (Chicken)	Abcam	AB4680	1:1000
#NeuN (Rabbit)	Millipore	ABN78	1:1000
#vGlut2 (Rabbit)	Abcam	AB101756	1:1000
#TH (Mouse)	Millipore	MAB318	1:200
#Nestin (Rabbit)	Millipore	ABD69	1:1000
#GFAP (Rabbit)	Abcam	AB7260	1:1000
#HNU (Mouse)	Millipore	MAB1281	1:200
Oct4 (Mouse)	Santa Cruz	SC-5279	1:500
Sox2 (Rabbit)	Cell signaling	#3579	1:500
SSEA4 (Mouse)	Millipore	MAB4304	1:500
Tra1-60 (Mouse)	Millipore	MAB4360	1:500
Tra1-81 (Mouse)	Millipore	MAB4381	1:500
Nestin (Rabbit)	Millipore	MAB5326	1:1000
TUJ1 (Mouse)	Covance	MMS-435P	1:1000
SMA (Mouse)	AbFrontier	YF-PA23164	1:500
PECAM (Mouse)	Millipore	MAB1393	1:500
AFP (Mouse)	Abcam	AB3980	1:500
FoxA2 (Mouse)	AbFrontier	YF-MA10439	1:500
Pax6 (Chicken)	DSHB	Kawakami, A.	1:200
Sox1 (Rabbit)	Millipore	AB15766	1:200
#NG2 (Rabbit)	Millipore	AB5320	1:200
#Olig2 (Rabbit)	Abcam	AB42453	1:200

#: Fluorescence staining was performed overnight.
