# Supplementary material for: Human-induced pluripotent stem cells generated from intervertebral disc cells improve neurologic functions in spinal cord injury
Source: Stem Cell Res Ther. 2015 Jun 24;6(1):125. doi: 10.1186/s13287-015-0118-x (PMC4529688; doi:10.1186/s13287-015-0118-x)
Supplement: Additional file 2: Table S4. — List of primer pairs used in this study. [file 13287_2015_118_MOESM2_ESM.pdf]

Table 4. List of primer pairs used in this study

	Gene	Forward primer (5' – 3')	Reverse primer (5' – 3')
Real-time PCR	***Oct4***	agaagaggatcaccctgggat	agaaccacactcggaccacat
	DNMT3B	gctcacagggcccgatactt	gcagtcctgcagctcgagttta
	Nanog	cctgaagacgtgtgaagatg	gctgattaggctccaaccat
	Zic3	caaagtgtgcgacaagtcctaca	gggaggaatctgacccttgag
	REX1	cagatcctaaacagctcgcag	gcgtacgcaaattaaagtcca
	Sox1	cgccgagttgagcaaga	ttcagccgcttcacctg
	PAX6	gcttcaccatggcaaataacc	ggcagcatgcaggagtatga
	GATA2	cagacgacaaccaccaccttatg	tggtcagtggcctgttaacattg
	Brachyury	acccagttcatagcggtgac	ccattgggagtacccaggtt
	AFP	ttgggctgctcgctatg	tttgtaactgttgctgcctt
	SOX17	tggcgcagcagaatcca	ccacgacttgcccagcat
	GAPDH	gtggacctgacctgccgtct	ggaggagtgggtgtcgctgt
RT-PCR	***Aggrecan***	tctaccgctgcgaggtgat	tgtaatggaacacgatgccttt
	SOX9	ctgagcagcgacgtcatctc	gttgggcggcaggtactg
	COL2A1	ggaagagtggagactactggattgac	tccatgttgcagaaaaccttca
	Sox1	caatgcggggaggagaagtc	ctctggaccaaactgtggcg
	Pax6	ggcaacctacgcaagatggc	tgagggctgtgtctgttcgg
	PLZF	ctatgggcgagaggagagtg	tcaatacagcgtcagccttg
	DACH1	gtggaaaacacccctcagaa	cttgttccacattgcacacc
	PLAGL1	gcctcagtcacctcaaaagc	cttaccctgtggggcaaaga
	NR2F	acaggaactgtcccatcgac	gatgtagccggacaggtagc
	Endo Oct4	gacaggggggaggggaggagctagg	cttccctccaaccagttgccccaaac
	Endo Sox2	gggaaatgggaggggtgcaaaagagg	ttgcgtgagtgtggatgggattggtg
	Endo Klf4	acgatcgtggccccggaaaaggacc	tgattgtagtgctttctggctgggctcc
	Endo c-MYC	gcgtcctgggaagggagatccggagc	ttgaggggcatcgtcgcgggaggctg
	Trans Oct4	ccccagggccccattttggtacc	attttatcgtcgaccactgtgctg
	Trans Sox2	ggcacccctggcatggctcttggctc	attttatcgtcgaccactgtgctg
	Trans Klf4	acgatcctggccccggaaaaggacc	attttatcgtcgaccactgtgctg
	Trans c-MYC	caacaaccgaaaatgcaccagccccag	attttatcgtcgaccactgtgctg
	b-Actin	atctggcaccacaccttctac	gcgtacagggatagcacagc
Bisulfite sequencing	***Oct4***	ggatgttattaagatgaagatagttgg	cctaaactccccttcaaaatctatt
	Nanog	agagataggagggtaagttttttt	actcccacacaaactaacttttattc

Endo: endogenous; Trans: transgene
