# Supplementary material for: Human-induced pluripotent stem cells generated from intervertebral disc cells improve neurologic functions in spinal cord injury
Source: Stem Cell Res Ther. 2015 Jun 24;6(1):125. doi: 10.1186/s13287-015-0118-x (PMC4529688; doi:10.1186/s13287-015-0118-x)
Supplement: Additional file 3: Table S1. — DNA fingerprint analysis. [file 13287_2015_118_MOESM3_ESM.pdf]

Table 1. DNA fingerprint analysis

Locus / sample	Disc cell	diPSC1
D8S1179	12,14	12,14
D21S11	28,31	28,31
D7S820	8,11	8,11
CSF1PO	9,11	9,11
D3S1358	15,17	15,17
TH01	9,9	9,9
D13S317	9,11	9,11
D16S539	12,12	12,12
D2S1338	18,24	18,24
D19S433	15,15.2	15,15.2
vWA	18,18	18,18
TPOX	8,8	8,8
D18S51	12,15	12,15
D5S818	10,10	10,10
FGA	22,22	22,22
