# Supplementary material for: Human-induced pluripotent stem cells generated from intervertebral disc cells improve neurologic functions in spinal cord injury
Source: Stem Cell Res Ther. 2015 Jun 24;6(1):125. doi: 10.1186/s13287-015-0118-x (PMC4529688; doi:10.1186/s13287-015-0118-x)
Supplement: Additional file 4: Table S2. — Lists of human embryonic stem cell (hESC)-enriched genes and disc cell-enriched genes shown in Fig. 2j (left). [file 13287_2015_118_MOESM4_ESM.pdf]

Table 2. Lists of hESC-enriched genes and disc cell-enriched genes shown in Fig.2J (*left*)

hESC-enriched genes	GeneBank	Disc cell-enriched genes	GeneBank
ACTA1	NM_001100.3	AEBP1	NM_001129.3
AIF1L	NM_031426.2	AKR1C2	NM_001354.4
APOE	NM_000041.2	ALDH3A1	NM_000691.3
BEX1	NM_018476.3	ALPK2	NM_052947.3
BEX2	NM_032621.2	ANPEP	NM_001150.1
C9orf135	NM_001010940.1	APCDD1L	NM_153360.1
CACHD1	NM_020925.2	APPL2	NM_018171.3
CAMKV	NM_024046.3	ARID5B	NM_032199.1
CCND2	NM_001759.2	ASAP2	NM_003887.2
CD24	NM_013230.2	AXL	NM_021913.2
CDH1	NM_004360.2	BAPX1	NM_001189.2
CDH3	NM_001793.3	C10orf116	NM_006829.2
CNTNAP2	NM_014141.4	C14orf78	XM_001132404.1
CRMP1	NM_001014809.1	CAMK2N1	NM_018584.5
CXADR	NM_001338.3	CAV1	NM_001753.3
CYP2S1	NM_030622.6	CAV2	NM_001233.3
DPPA4	NM_018189.3	CD44	NM_001001392.1
EDNRB	NM_000115.1	CEBPD	NM_005195.3
EPCAM	NM_002354.2	COL16A1	NM_001856.3
FLJ40504	NM_173624.1	COL6A2	NM_001849.3
GPC4	NM_001448.2	COL8A1	NM_020351.2
GPM6B	NM_001001995.1	CPA4	NM_016352.2
HAND1	NM_004821.1	CYBRD1	NM_024843.2
HERC5	NM_016323.2	DAB2	NM_001343.2
IGF2BP3	NM_006547.2	DCN	NM_133505.2
IGFBP2	NM_000597.2	EFEMP2	NM_016938.2
KIF1A	NM_004321.4	FAM129B	NM_001035534.1
L1TD1	NM_019079.2	FAM20C	NM_020223.2
LDB2	NM_001290.2	FAM38A	NM_014745.1
LECT1	NM_007015.2	FBLN5	NM_006329.2
LIN28	NM_024674.4	FER1L3	NM_013451.2
LIN28B	NM_001004317.2	FGFRL1	NM_021923.3
LOC642559	XR_016333.1	FOXC1	NM_001453.1
LOC643272	XM_926633.1	FRMD6	NM_152330.2
LOC645682	XR_017655.1	GAS6	NM_000820.1

LRRN1	NM_020873.5	GAS6	NM_000820.1
MT1H	NM_005951.2	GREM1	NM_013372.5
MYCN	NM_005378.4	HERC4	NM_022079.2
NLGN4X	NM_020742.2	HTRA1	NM_002775.3
NNAT	NM_181689.1	IGFBP3	NM_001013398.1
NTS	NM_006183.3	IGFBP6	NM_002178.2
PODXL	NM_001018111.2	IGFBP7	NM_001553.1
POU5F1	NM_002701.4	ITGA3	NM_002204.1
POU5F1P1	NR_002304.1	KCNMA1	NM_002247.2
POU5F1P1	NR_002304.1	KDELR3	NM_016657.1
PPP2R2B	NM_181676.1	KDELR3	NM_006855.2
PROM1	NM_006017.1	LMNA	NM_005572.3
RASL11B	NM_023940.2	LOC399959	NR_024430.1
RPRM	NM_019845.2	LOC645638	XR_040455.1
SALL4	NM_020436.2	LOXL3	NM_032603.2
SBK1	NM_001024401.2	LPAR1	NM_057159.2
SEMA6A	NM_020796.3	LTBR	NM_002342.1
SFRP2	NM_003013.2	LXN	NM_020169.2
SLC7A3	NM_032803.4	MALL	NM_005434.3
SOX2	NM_003106.2	MVP	NM_005115.3
TACSTD1	NM_002354.1	MXRA5	NM_015419.2
TNFRSF21	NM_014452.3	MYOF	NM_013451.3
UCA1	NR_015379.2	NDRG1	NM_006096.2
ZFP42	NM_174900.3	OBFC1	NM_024928.3
ZIC2	NM_007129.2	PAPSS2	NM_004670.3
ZIC3	NM_003413.2	PCOLCE	NM_002593.2
ZSCAN10	NM_032805.1	PDGFRB	NM_002609.3
		PPP1R3C	NM_005398.4
		PTGER2	NM_000956.2
		RPS6KA2	NM_001006932.1
		S100A16	NM_080388.1
		S100A4	NM_019554.2
		SCARA3	NM_016240.2
		SIRPA	NM_001040023.1
		SLFN11	NM_152270.2
		STEAP3	NM_018234.2
		TM4SF1	NM_014220.2
		TMEM166	NM_032181.1

		TSPO	NM_007311.3
		ZAK	NM_133646.2
