# Supplementary material for: Human-induced pluripotent stem cells generated from intervertebral disc cells improve neurologic functions in spinal cord injury
Source: Stem Cell Res Ther. 2015 Jun 24;6(1):125. doi: 10.1186/s13287-015-0118-x (PMC4529688; doi:10.1186/s13287-015-0118-x)
Supplement: Additional file 5: Table S3. — Lists of human embryonic stem cell (hESC)-enriched genes and chondrogenic genes shown in Fig. 2j (right). [file 13287_2015_118_MOESM5_ESM.pdf]

Table 3. Lists of hESC-enriched genes and chondrogenic genes shown in Fig.2J (*right*)

hESC-enriched genes	GeneBank	Chondrogenic genes	GeneBank
DPPA2	NM_138815.2	COL2A1	NM_001844.3
ZIC3	NM_003413.2	PAX1	NM_006192.1
TERT	NM_198253.2	COL1A2	NM_000089.3
DPPA4	NM_018189.3	SOX9	NM_000346.2
POU5F1	NM_002701.4	COL1A1	NM_000088.3
LIN28	NM_024674.4	SOX9	NM_000346.2
GDF3	NM_020634.1	FOXF1	NM_001451.2
SOX2	NM_003106.2		
NANOG	NM_024865.2		
